# Anti-inflammatory and vasculogenic conditioning of peripheral blood mononuclear cells reinforces their therapeutic potential for radiation-injured salivary glands

**DOI:** 10.1186/s13287-019-1414-7

**Published:** 2019-10-17

**Authors:** Takashi I., Yoshinori Sumita, Takako Yoshida, Ryo Honma, Mayumi Iwatake, Jorge Luis Montenegro Raudales, Tomoko Shizuno, Shinichiro Kuroshima, Haruchika Masuda, Makoto Seki, Simon D. Tran, Takayuki Asahara, Izumi Asahina

**Affiliations:** 1 Department of Regenerative Oral Surgery, Unit of Translational Medicine, Nagasaki University Graduate School of Biomedical Sciences, Nagasaki, Japan; 2 Basic and Translational Research Center for Hard tissue Disease, Nagasaki University Graduate School of Biomedical Sciences, 1-7-1 Sakamoto, Nagasaki, 852-8588 Japan; 3 CellAxia Inc., Tokyo, Japan; 4 Department of Applied Prosthodontics, Nagasaki University Graduate School of Biomedical Sciences, Nagasaki, Japan; 5 Department of Regenerative Medicine Science, Tokai University School of Medicine, Isehara, Japan; 6 Laboratory of Craniofacial Tissue Engineering and Stem Cells, Faculty of Dentistry, McGill University, Montreal, Canada

**Keywords:** Radiogenic salivary gland dysfunction, Cell-based therapy, Peripheral blood mononuclear cells, Macrophage, Anti-inflammation, Vasculogenesis

## Abstract

**Background:**

There are currently no effective treatments available for patients with irreversible loss of salivary gland (SG) function caused by radiation therapy for head and neck cancer. In this study, we have developed an effective culture method to enhance the anti-inflammatory and vasculogenic phenotypes of peripheral blood mononuclear cells (PBMNCs) and investigated whether such effectively conditioned PBMNCs (E-MNCs) could regenerate radiation-injured SGs and ameliorate salivary secretory function in mice.

**Methods:**

Mouse PBMNCs were expanded in primary serum-free culture with five vasculogenic proteins for 5 days, and then the resulting cells (E-MNCs) were analyzed for their characteristics. Subsequently, 5 × 10^4^ E-MNCs (labeled with EGFP in some experiments) were injected intra-glandularly into a mouse model of radiation-injured atrophic submandibular glands. After 2–3 weeks, the submandibular glands were harvested, and then the injected E-MNCs were tracked. Four, 8, and 12 weeks after irradiation (IR), salivary outputs were measured to evaluate the recovery of secretory function, and the gland tissues were harvested for histological and gene expression analyses to clarify the effects of cell transplantation.

**Results:**

The resulting E-MNCs contained an enriched population of definitive CD11b/CD206-positive (M2 macrophage-like) cells and showed anti-inflammatory and vasculogenic characteristics. Salivary secretory function in E-MNC-transplanted mice gradually recovered after 4 weeks post-irradiation (post-IR) and reached 3.8-fold higher than that of non-transplanted mice at 12 weeks. EGFP-expressing E-MNCs were detected in a portion of the vascular endothelium and perivascular gland tissues at 2 weeks post-IR, but mainly in some microvessels at 3 weeks. Between 4 and 12 weeks post-IR, mRNA expression and histological analyses revealed that E-MNC transplantation reduced the expression of inflammatory genes and increased the level of tissue-regenerative activities such as stem cell markers, cell proliferation, and blood vessel formation. At 12 weeks post-IR, the areas of acinar and ductal cells regenerated, and the glands had less fibrosis.

**Conclusions:**

This effective conditioning of PBMNCs is a simple, rapid, and efficient method that provides a non-invasive source of therapeutic cells for regenerating radiation-injured atrophic SGs.

## Background

Many patients with head and neck cancer are treated with radiotherapy either alone or in combination with surgery and/or chemotherapy. After such treatment, although the patient’s prognosis is improved, the radiogenic damage to the salivary glands (SGs) frequently causes severe salivary hypofunction. These patients suffer from severe xerostomia, dysphagia, dental caries, oropharyngeal infections, and oral mucositis [1, 2], and such complications lead to the reduction in quality of life [3]. To reduce radiation damage to SGs, approaches such as brachytherapy, intensity-modulated radiation therapy (IMRT), and heavy particle beam therapy have shown merit, but a significant proportion of patients still experience salivary hypofunction [4]. There are currently no adequate treatments for patients with such irreversible glandular damage. Therefore, developing an adequate treatment is urgently needed for the restoration of radiation-induced atrophic SGs.

For functional restoration of damaged SGs due to irradiation, various experimental approaches, such as the use of gene therapy, tissue engineering, and cell-based therapy, have been investigated [5–8]. In particular, cell-based therapies for atrophic SG using intra-glandular or intra-venous injections of SG stem/progenitor cells or mesenchymal stem cells (MSCs) have succeeded in slowing the atrophic process [9, 10]. For instance, it has been shown that spheroid cultures of adult SG cells can enrich progenitor cells (salispheres) and that intra-glandular transplantation of salisphere-derived c-kit^+^-expressing cells partially restores the tissue morphology and organ function of radiation-damaged SGs [11]. Likewise, we have previously focused on bone marrow-derived cells (BMDCs) including MSCs and found that BMDCs or cultured MSCs can improve the function of damaged SGs in both irradiation and Sjögren’s syndrome mouse models [6, 12]. These cells display paracrine effects by releasing anti-inflammatory, vasculogenic, or anti-apoptotic cytokines such as IL-10, VEGF, bFGF, and HGF to damaged glands [13, 14]. These experimental approaches strongly suggest that cell-based therapies are promising as an alternative remedy. However, the cell sources reported to date such as SG, bone marrow, adipose, and dental pulp tissues [6, 9, 11, 15] require invasive procedures to harvest at the donor site, particularly for elderly patients. In addition, securing a sufficient number of highly functional stem/progenitor cells in culture to ensure their therapeutic effects remains a challenge.

Meanwhile, it has been demonstrated that autologous human CD34^+^ and CD133^+^ stem/progenitor cells in bone marrow (BM) or peripheral blood (PB) have therapeutic effects, including neovascularization in patients with severe ischemic diseases [16]. These stem/progenitor cells serve as an enriched source of endothelial progenitor cells (EPCs). It is well known that EPCs favor angiogenesis and vasculogenesis by their proliferation and differentiation abilities [16]. In fact, a cell-based therapy using EPCs strongly promotes the revascularization and restoration of numerous ischemic tissues, including the myocardium, brain, and skin [17–19]. This indicates that blood vessel formation and blood supply are essential for tissue regeneration. However, EPC-enriched populations (CD34^+^, CD133^+^, CD34^+^/VEGFR-2^+^, or CD133^+^/VEGFR-2^+^) are usually scarce even in BM and PB, and they decline numerically and functionally in patients with aging or morbidity [20]. Therefore, to augment the qualitative and/or quantitative properties of such vasculogenic cell fractions, we have recently developed a novel culture method called “quality- and quantity-controlled culture (QQ-culture)” of BM-, PB-, and umbilical cord blood MNCs [21]. This serum-free QQ-culture system contains five recombinant proteins (stem cell factor, thrombopoietin, vascular endothelial growth factor, interleukin-6, and Flt-3 ligand) and can successfully enhance the vasculogenic potential of BMMNCs and PBMNCs in primary culture. Indeed, QQ-culture can reverse the diabetic vasculogenic dysfunction of BMMNCs and achieve the control levels of EPC function [22]. We have also shown the potential of cell-based therapy using human QQ-cultured PBMNCs for ischemic diseases [23]. These results have implications for the use of QQ-cultured PBMNCs to ameliorate ischemic salivary dysfunction, for which no adequate treatments are currently available. However, interestingly, QQ-culture of PBMNCs promotes not only the expansion of EPCs but also the adoption of regenerative phenotypes by macrophages and T lymphocytes [23]. In particular, the M2 phenotype of macrophage-like cells rather than EPCs appears at a specific period (5–7 days) of this primary culture. Therefore, we assumed that this specific QQ-culture condition (which we named “5G-culture”) of PBMNCs may be extremely effective in promoting tissue regenerative activity with a phenotype transition between anti-inflammatory macrophages and T lymphocytes. It has been shown that paracrine effects by MSC therapy function via induction of the anti-inflammatory phenotype of activated macrophages at injured sites, and such macrophages contribute to neovascularization and subsequent tissue regeneration [24–26].

The aim of this study was to investigate whether effectively conditioned PBMNCs (effective PBMNCs; E-MNCs), resulting from the induction of M2 macrophages, could rescue radiation-induced SG hypofunction in mice by direct transplantation of cells into the submandibular glands. This study is a pre-requisite step for future clinical trials aimed at developing cell-based therapies for atrophic SGs. Although the mechanisms of regeneration are not currently well understood, we have previously found that autologous BMDC injections have beneficial therapeutic effects via the promotion of anti-inflammation and vasculogenesis in radiogenic-injured SGs or oral mucositis mouse models [6, 27]. Therefore, the use of E-MNCs, which do not require a long cell expansion process, is a simple and direct approach using a readily available source of cells (blood) that can be obtained with low invasiveness.

## Methods

### Animals

C57BL/6JJcl mice (inbred strain) (CLEA Japan Inc., Tokyo, Japan) were employed as the recipient (female mice) and donors (male mice), and C57BL/6-Tg (CAG-EGFP) male mice (Japan SLC Inc., Shizuoka, Japan) were used in some experiments as donors. All mice were kept under clean conventional conditions at the Nagasaki University animal center. All experimental procedures were performed in accordance with the guidelines approved by the Nagasaki University Ethics Committee (1605271307 and 1610051411).

### Serum-free 5G-culture of PBMNCs

Mouse PBMNCs were cultured under the 5G-culture condition, which is a modified human QQ-culture system as described in our previous work [23], specific conditions of which were established by CellAxia Inc. (Tokyo, Japan). Briefly, peripheral blood was obtained by collecting the blood from the hearts of donor mice, and the fraction containing PBMNCs was isolated by density gradient centrifugation using separating medium (Histopaque-1083; Sigma Aldrich, St. Louis, MO, USA). Then, the isolated PBMNCs were seeded on 6-well Primaria tissue culture plates (BD Biosciences, San Jose, CA, USA) at a density of 2 × 10^6^ cells/2 ml of 5G-culture medium per well, and cultured for 5 days in serum-free 5G-culture medium (Stemline II Hematopoietic Stem Cell Expansion Medium; Sigma Aldrich), which was supplemented with five mouse recombinant proteins (Table 1). After 5 days of culture, 5G-cultured cells (effectively conditioned PBMNCs; E-MNCs) were harvested for subsequent experiments. In order to track the donor cells post-transplantation, some E-MNCs were obtained from C57BL/6-Tg (CAG-EGFP) mice.

**Table 1:** Contents of 5G-culture medium

Contents	Company, catalog no.	Final concentration (ng/mL)
rm SCF	Peprotech, #250-03	100
rm flt-3 ligand	Peprotech, #250-31 L	100
rm TPO	Peprotech, #315-14	20
rm VEGF	Peprotech, #450-32	50
rm IL-6	Peprotech, #216-16	20

### Evaluation of the characteristics of E-MNCs: flow cytometry analysis

Freshly isolated PBMNCs and E-MNCs were subjected to flow cytometry to detect the surface antigen positivity of macrophage subpopulations [M1: CD11b^+^/CD206^−^; M2: CD11b^+^/CD206^+^ and CD11b^+^/CCR2^−^/Galectin3^+^ [28]], T lymphocytes (CD3^+^) [Th1: CXCR3^+^; Th2: CXCR4^+^/CXCR6^−^; Th17: CXCR4^+^/CXVR6^+^ in CD3^+^/CD4^+^ T helper cells], B lymphocytes (B220^+^), and endothelial stem/progenitor cells (c-Kit^+^/Sca-1^+^/lineage^−^), along with the cell viability (propidium iodide (PI)). The Abs used are listed in Table 2. Cells were suspended in 2 mmol/l of ethylenediaminetetraacetic acid (EDTA/0.2% bovine serum albumin (BSA)/phosphate-buffered saline (PBS) buffer (5 × 10^5^ cells/200 μl), were incubated after the addition of 10 μl of FcR blocking reagent at 4 °C for 30 min, and then equally dispensed into reaction tubes for subsequent staining (100 μl/tube). Each aliquot was incubated with 2 μl of each 1st-Ab at 4 °C for 20 min and then washed twice with 1 ml of 2 mmol/l EDTA/0.2% BSA/PBS buffer. Cells were resuspended in 2 mmol/l of EDTA/0.2% BSA/PBS buffer (2 × 10^5^ cells/200 μl). Flow cytometry analysis was performed using a LSRFortessa cell analyzer (BD Biosciences) and FlowJo software (Tomy Digital Biology Co., Ltd., Tokyo). The percent positivity of macrophage subpopulations and endothelial stem/progenitor cells per each gate in PBMNCs or E-MNCs was evaluated and then calculated in relation to that of the whole cell population.

**Table 2:** Antibodies for flow cytometry

Antibodies for flow cytometry	Company, catalog no.
APC anti-mouse CD206 (MMR)	Biolegend, #141707
APC rat IgG2a, κ isotype Ctrl	Biolegend, # 400511
APC-Cy7 anti-mouse/human CD11b	Biolegend, #101225
APC/Cy7 anti-mouse CD4	Biolegend, #100413
APC/Cy7 rat IgG2b, κ isotype Ctrl	Biolegend, #400623
PE-Cy7 anti-mouse/human Mac-2 (Galectin-3)	Biolegend, #125417
PE-Cy7 rat IgG2a, κ isotype Ctrl	Biolegend, #400521
APC anti-mouse CCR2-conjugate	R&D, #FAB5538A
APC rat IgG2b, κ isotype Ctrl	Biolegend, #400611
FITC anti-mouse/human CD45R/B220	Biolegend, #103205
FITC rat IgG2a, κ isotype Ctrl	Biolegend, #400505
PE anti-mouse CD3	Biolegend, #100205
PE rat IgG2b, κ isotype Ctrl	Biolegend, #400607
FITC anti-mouse CD183 (CXCR3)	Biolegend, #126535
FITC Armenian hamster IgG, isotype Ctrl	Biolegend, #400905
PE/Cy7 anti-mouse CD194 (CCR4)	Biolegend, #131213
PE/Cy7 Armenian hamster IgG, isotype Ctrl	Biolegend, #400921
APC anti-mouse CD196 (CCR6)	Biolegend, #129813
APC Armenian hamster IgG, isotype Ctrl	Biolegend, #400911
True-Stain Monocyte Blocker™	Biolegend, #426101
PE-Cy7 labeled mouse anti-mouse CD117 (c-Kit)	Biolegend, #135111
PE-Cy7 rat IgG2b, κ isotype Ctrl	Biolegend, #400617
APC-Cy7 labeled mouse anti-mouse Ly-6A/E (Sca-1)	Biolegend, #108125
APC-Cy7 rat IgG2b, κ isotype Ctrl	Biolegend, #400523
Lineage cocktail; biotin rat anti-mouse CD45R/B220	BD, #553086
Lineage cocktail; biotin rat anti-mouse TER-119/erythroid cells	BD, #553672
Lineage cocktail; biotin Armenian hamster anti-mouse CD3e	BD, #553060
Lineage cocktail; biotin rat anti-mouse CD11b antibody	BD, #553309
Lineage cocktail; biotin rat anti-mouse Ly-6G/Ly-6c	BD, #553124
Streptavidin-FITC conjugate	BD, #553141
PI	WAKO, #169-26281

### Evaluation of the characteristics of E-MNCs: anti-inflammatory and vasculogenic gene expressions

Quantitative real-time PCR was used to determine the mRNA expressions of anti-inflammatory and vasculogenic (IL-1β, IFN-γ, IL-10, VEGF-A, and B) genes in PBMNCs and E-MNCs; each was measured in triplicate. Total RNA was extracted with Trizol reagent (Invitrogen, Waltham, MA, USA), and first-strand complementary DNA synthesis was performed using SuperScript First-Strand Synthesis (Invitrogen). Complementary DNA was amplified with Takara-Taq (Takara Bio Inc., Shiga, Japan). PCR reactions were performed with an Mx3000P QPCR System (Agilent technology). Mouse-specific primer sets are shown in Table 3. As an internal standard, glyceraldehyde-3-phosphate dehydrogenase (*gapdh*) was used.

**Table 3:** Mouse primer sets

Gene	Forward primer	Reverse primer
*il-1β*	5′-GCTGAAAGCTCTCCACCTCA-3′	5′-AGGCCACAGGTATTTTGTCG-3′
*Il-10*	5′-GCTGGACAACATACTGCTAACC-3′	5′-ATTTCCGATAAGGCTTGGCAA-3′
*ifn-γ*	5′-ACAGCAAGGCGAAAAAGGATG-3′	5′-TGGTGGACCACTCGGATGA-3′
*tnf-α*	5′-TACTTAGACTTTGCGGAG-3′	5′-AGAGTAAAGGGGTCAGAG-3′
*vegf-a*	5′-CCTCCGAAACCATGAACTTT-3′	5′-TCATGGGACTTCTGCTCTCC-3′
*vegf-b*	5′-CTCATGATCCAGTACCCGAGC-3′	5′-GCTTCACAGCACTCTCCTTT-3′
*vegf-c*	5′-CTCAATGCATGCCACGTGAG-3′	5′-CAGCAACCCCCACATCTGTA-3′
*flt-1*	5′-CTCACTTGCACCGTGTATGG-3′	5′-TGCTGGGATCCAGGATAAAG-3′
*flt-4*	5′-GACGAGCTGGTGAAGCTACC-3′	5′-TCACCTCTTTGAGCACCAGA-3′
*flk-1*	5′-GCTTGCCTTATGATGCCAGC-3′	5′-TCCAAAAGCGTCTGCCTCAA-3′
*Aqp5*	5′-CCTTATCCATTGGCTTGTCG-3	5′-CCCAGAAGACCCAGTGAGAG-3
*gapdh*	5′-TGCACCACCAACTGCTTAG-3′	5′-GGATGCAGGGATGATGTTC-3′

### Evaluation of characteristics of E-MNCs: EPC colony-forming assays

**Fig. 1:**
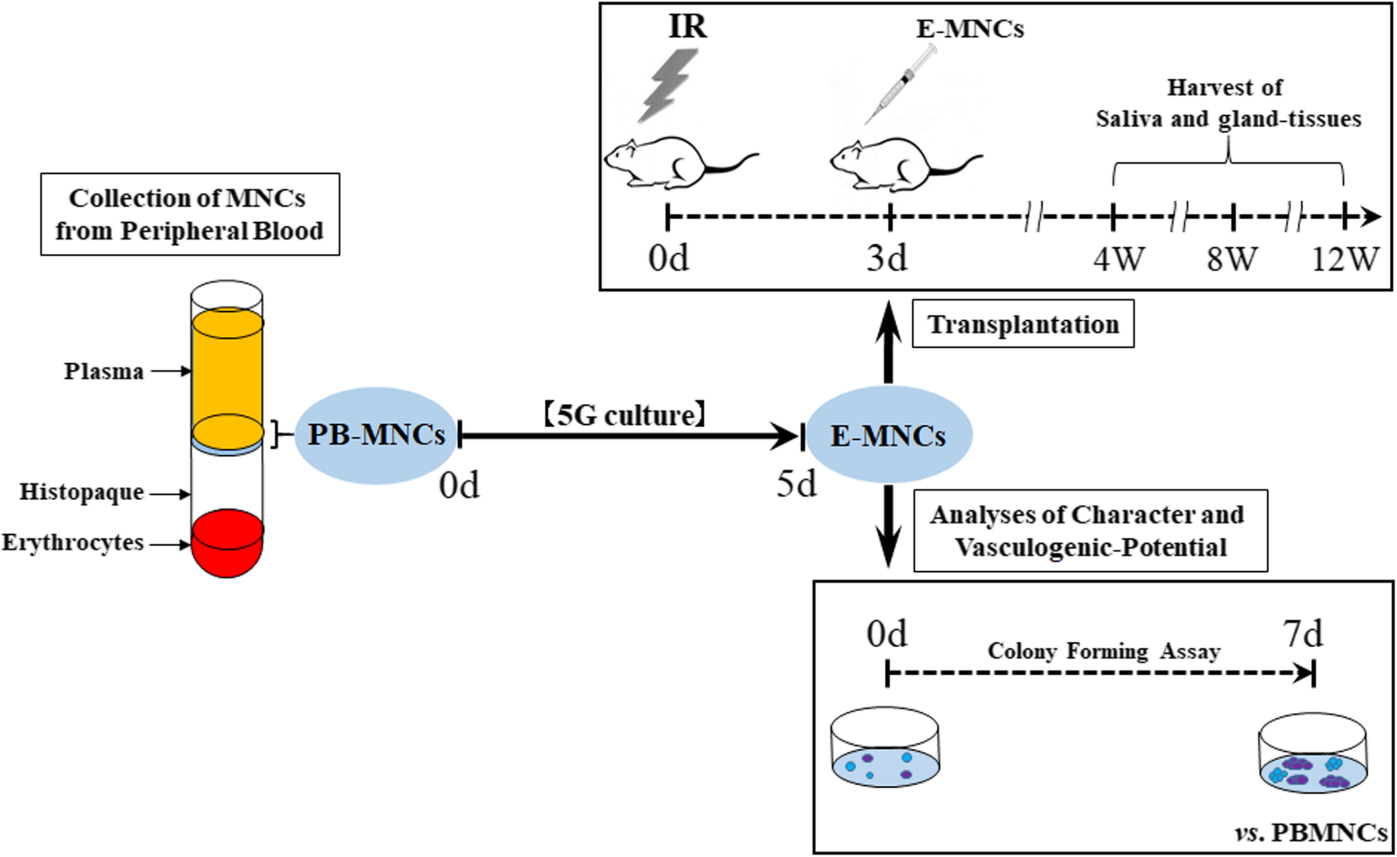
Schematic diagram describing the experimental design for E-MNC-based therapy of radiogenic-injured salivary glands

To investigate the vasculogenic potential of PBMNCs (pre-5G-culture) and E-MNCs (post-5G-culture), the cells were seeded in 35-mm Primaria dishes (BD Biosciences) at 1 × 10^5^ cells/dish (three dishes per culture), and then EPC colony-forming assays (EPC-CFA) were performed by using semisolid culture medium (MethoCult SF^BIT^; STEMCELL Technologies, Inc., Vancouver, Canada) with pro-angiogenic growth factors/cytokines (Fig. 1, Table 4) as we previously reported [22]. Seven days after initiation of EPC-CFA, the number of adherent colonies per dish was assessed under a microscope, and the EPC colony-forming units (EPC-CFUs) were counted as previously described [21]. Simultaneously, to confirm the endothelial characteristics of colonized cells, we assessed the biochemical binding of isolectin B4-conjugated fluorescein isothiocyanate (ILB4-FITC) (Vector Laboratories, Burlingame, CA, USA), which is a marker of endothelial cells, and the uptake of acetylated low-density lipoprotein labeled with 1,1′-dioctadecyl-3,3,3′,3′-tetramethylindo-carbocyanine perchlorate (AcLDL-DiI) (Biomedical Technologies, Inc., Stoughton, MA, USA), which is metabolized by a receptor-mediated process in endothelial cells, by fluorescence microscopy.

**Table 4:** Contents in semisolid culture for EPC-CFA

Contents	Company, catalog no.	Final concentration
rm VEGF	Peprotech, #450-32	50 ng/mL
rm basic FGF	Peprotech, #450-33	50 ng/mL
rm EGF	Peprotech, #315-09	50 ng/mL
rm IGF-1	Peprotech, #250-19	50 ng/mL
rm SCF	Peprotech, #250-03	100 ng/mL
rm IL-3	Peprotech, #213-13	20 ng/mL
Heparin	YOSHINDO, #121-154	2 U/mL
FBS	SAFC Biosciences, #12303	30%

### Irradiation (IR) of mice and transplantation of E-MNCs

C57BL/6 mice were anesthetized with 10 μl/g body of ketamine (10 mg/ml) given by intraperitoneal (ip) injection and restrained in a container for irradiation. The submandibular glands were damaged by exposing them to a single dose of 12-Gy using gamma rays from PS-3100SB (Pony Industry Co, Ltd., Osaka, Japan). The radiation was collimated to the head and neck area to guarantee less than 10% beam strength in the rest of the body. Then, E-MNCs (5 × 10^4^ cells per gland, a total of 1 × 10^5^ cells) were directly injected into the submandibular glands at 3 days post-IR (Fig. 1). For transplantation, mice were randomly divided into four groups in a blinded fashion: (1) non-irradiation and non-cell transplantation [control group; *n* = 5 for each time point, sacrificed at 1, 2, 3, 4, 8, and 12 weeks after IR), total *n* = 30], (2) irradiation and injection of 20 μl IMDM (Iscove’s modified Dulbecco’s media; Sigma Aldrich) without cell transplantation [sham group as an experimental control; *n* = 5 at each time point (4, 8, and 12 weeks after IR), total *n* = 15], (3) irradiation and transplantation of E-MNCs with 20 μl IMDM [E-MNC group; *n* = 5 at each time point (2, 3, 4, 8, and 12 weeks after IR), total *n* = 25], and (4) irradiation and transplantation of PBMNCs with 20 μl IMDM [PBMNC group; *n* = 5 at each time points (4, 8, and 12 weeks after IR), total *n* = 15].

### Salivary flow rate and gross appearance of SGs

To measure the secretory function (salivary flow rate (SFR)) of SGs, mice were kept under general anesthesia by ip injection of 10 μl/g body weight of ketamine. Whole saliva was collected after stimulation of secretion by 0.5 mg/kg body weight pilocarpine (Sigma Aldrich) administered subcutaneously. Saliva was obtained from the oral cavity by a micropipette and placed into pre-weighed 1.5-ml microcentrifuge tubes. It was collected for a 10-min period and its volume determined gravimetrically. SFR was determined at weeks 0, 4, 8, and 12 post-IR, *n* = 5/group at each time point. At 2, 3, 4, 8, and 12 weeks after IR, the mice were sacrificed, and their submandibular glands were harvested. At that time, the body weights and the weights of submandibular glands were measured, and the gross appearance of harvested submandibular glands was observed before fixation, *n* = 5/group at each time point.

### Detection of transplanted E-MNCs

To track EGFP-expressing E-MNCs, the expression of green fluorescence in frozen sections was observed at weeks 2 and 3 post-IR in control and E-MNC groups. The blood vessels were stained by intravenous injection with ILB4-DyLight 594 (red) (Vector Laboratories) just before sacrificing. Five-micrometer sections were fixed in 4% paraformaldehyde (PFA), rinsed by 0.01 mol/l PBS, and the nuclei were counterstained with 4′,6-diamidino-2′-phenylindole (DAPI; Vector Laboratories, Burlingame, CA, USA). Then, they were examined by a confocal laser microscope (LSM 800 with Airyscan; Carl Zeiss, Inc., Oberkochen, Germany) at × 200 and × 400 magnification, *n* = 5/group at each time point. Additionally, two examiners independently counted the number of EGFP-expressing cells in the parenchyma and microvessels separately in a blinded manner under × 200 magnification, five different fields per specimen/five sections, *n* = 5/group at 2 and 3 weeks post-IR.

### Histological and immunohistological observations

The harvested submandibular glands were fixed in 4% PFA and embedded in paraffin. Five-micrometer sections were stained with hematoxylin and eosin (H&E) and examined microscopically under × 200 magnification, *n* = 5/group at each time point.

For Masson’s trichrome staining (Masson’s Trichrome Stain Kit; Agilent Technology, Santa Clara, CA, USA) after deparaffinization and hydration, staining was performed with Weigert’s iron hematoxylin solution and Biebrich scarlet-acid fuchsin. After that, tissues were stained with phosphotungstic/phosphomolybdic acid solution, aniline blue solution, and 1% acetic acid. Fibrosis was assessed by light microscopy under × 40 magnification via five random fields in a section for five sections/specimen. The percentage of fibrosis area was analyzed by ImageJ software (National Institutes of Health, Bethesda, MD, USA), *n* = 5/group at 12 weeks post-IR.

Furthermore, for periodic acid-Schiff (PAS) staining, tissue sections were analyzed using a PAS Kit (Sigma Aldrich). After deparaffinization and hydration, staining was performed with periodic acid solution and Schiff’s reagent. Then, sections were counterstained with hematoxylin solution. The percentage of surface area occupied by acinar cells was assessed by light microscopy under × 100 magnification and analyzed by ImageJ software via three different fields in a section/five sections, *n* = 5/group at 12 weeks post-IR.

Immunohistological staining was performed with rat anti-mouse SCFR/c-Kit/CD117 antibody (1:50; R&D Systems, Minneapolis, MN, USA), rabbit anti-mouse Sca-1/Ly6 antibody (1:50; Abcam, Cambridge, UK), rabbit anti-mouse CD31 antibody (1:50; Abcam), mouse anti-mouse pan-cytokeratin antibody (1:100; Abcam), and goat anti-mouse NKCC1 antibody (1:100; Santa Cruz Biotechnology, Santa Cruz, CA, USA). The slides were then incubated with Alexa Fluor 546-conjugated goat anti-rabbit antibody (1:100; Thermo Fisher Scientific Life Sciences, Waltham, MA, USA) for Sca-1 and CD31, FITC-conjugated donkey anti-rat antibody (1:100; Abcam) for c-Kit, TRITC-conjugated donkey anti-goat antibody (1:200; Abcam) for NKCC1, and FITC-conjugated donkey anti-mouse antibody (1:100; Abcam) for cytokeratin as secondary antibodies and counterstained with mounting medium for fluorescence with DAPI (Vector Laboratories). Sca-1/c-Kit, CD31/cytokeratin, and NKCC1 were examined by a fluorescence microscope under × 200 magnification. Then, two examiners independently counted the number of c-Kit/Sca-1-positive cells in a blinded manner via three different fields in a specimen/five sections, *n* = 5/group at 4 weeks post-IR. For the assessment of the blood vessels and ducts, the percentage of surface area occupied by the blood vessels (stained by CD31) or ducts (stained by cytokeratin) was analyzed by ImageJ software via three different fields in a specimen/five sections, *n* = 5/group at 4, 8, and 12 weeks post-IR.

For PCNA, tissues were stained with rat anti-mouse PCNA antibody (1:1000; Abcam) and the VECTASTAIN ABC Kit (Vector Laboratories). After that, they were counterstained with hematoxylin. Tissues were examined by a light microscope under × 200 magnification. Then, two examiners independently counted the number of positive cells in a blinded manner via three different fields in a specimen/five sections, *n* = 5/group at 4 weeks post-IR.

### Inflammatory, vasculogenic, and saliva production gene expressions

Quantitative real-time PCR was used to determine the mRNA expressions of inflammatory (IL-1β, IFN-γ, and TNF-α), vasculogenic (VEGF-A, VEGF-B, VEGF-C, Flt-1, Flt-4, and Flk-1), and saliva production (AQP5) genes in the submandibular glands, *n* = 3/group at 4 and 8 weeks post-IR. Total RNA was extracted with Trizol reagent, and first-strand complementary DNA synthesis was performed by SuperScript First-Strand Synthesis. Complementary DNA was amplified with Takara-Taq. PCR reactions were performed with an Mx3000P QPCR System. Mouse-specific primer sets are shown in Table 3. As an internal standard, glyceraldehyde-3-phosphate dehydrogenase (*gapdh*) was used.

### Epidermal growth factor (EGF) concentration

Concentration of EGF in saliva (*n* = 4/group in sham and E-MNC groups at week 8 post-irradiation) was measured by ELISA method (Abcam). This assay employed a quantitative sandwich enzyme immunoassay technique. The intensity of the color measured is in proportional to the amount of EGF. The sample values were compared to the EGF standard curve.

### Statistical analysis

Means were analyzed via one-way analysis of variance. The Dunnett multiple comparison *t* test was used to detect any significant differences within each group. Experimental values are presented as means ± SD; *p* < 0.05 was considered statistically significant.

## Results

### Characteristic changes in anti-inflammatory and vasculogenic potential of MNCs after 5G-culture

**Fig. 2:**
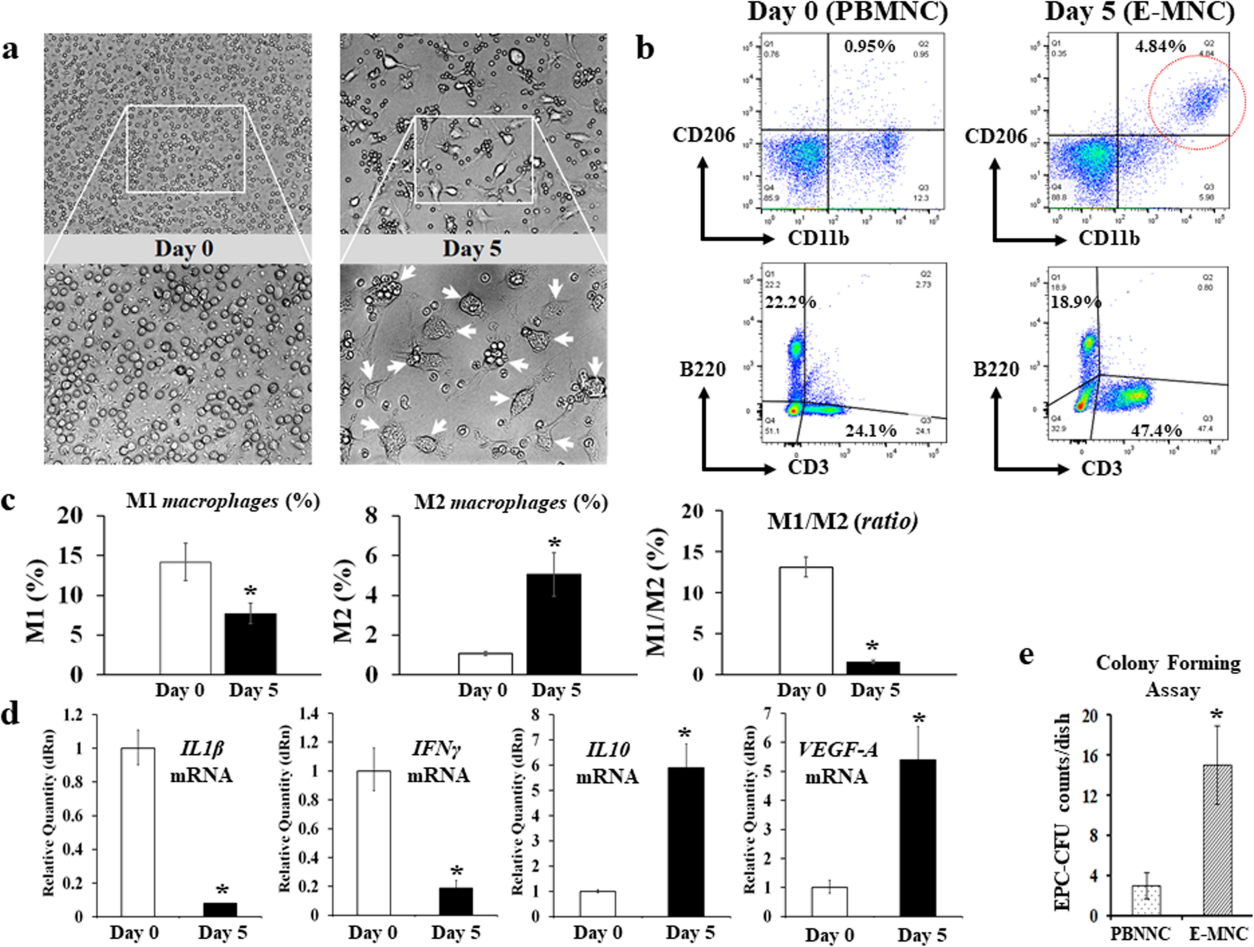
Changes in anti-inflammatory and vasculogenic potential of MNCs in 5G-culture. **a** Phase contrast imaging of PBMNCs (at day 0) and E-MNCs (at day 5). The white box areas in the upper images (× 100) were magnified in the lower images (× 200). **b** Flow cytometric analysis of M1 (CD11b^+^/CD206^−^) macrophages, M2 (CD11b^+^/CD206^+^) macrophages, T lymphocytes (CD3^+^), and B lymphocytes (B220^+^) fractions in PBMNCs (at day 0) and E-MNCs (at day 5). **c** Percentages of M1 (CD11b^+^/CD206^−^) and M2 (CD11b^+^/CD206^+^) macrophage fractions and the ratios (M1/M2) in PBMNCs and E-MNCs. **d** mRNA expressions of inflammatory genes (IL-1β and IFN-γ), an anti-inflammatory gene (IL10), and vasculogenic genes (VEGF-A) of PBMNCs (at day 0) and E-MNCs (at day 5) (*p < 0.05). **e** Counts of EPC-CFUs from pre-cultured PBMNCs and post-cultured PBMNCs (E-MNCs) per dish (1 × 10^5^ cells/dish)

At 5 days of culture, some adherent cells became larger and changed their morphology to macrophage-like round shapes (Fig. 2a). Then, flow cytometry revealed that an M2 macrophage-enriched fraction (CD11b^+^/CD206^+^) distinctly appeared in E-MNCs at this time (from 1.07 to 5.51%) (Fig. 2b, c). Furthermore, CCR2^−^/Galectin3^+^ cells (as M2 macrophages) in the CD11b-positive cell fraction obviously increased (from 3.08 to 57.1%) after 5G-culture (Additional file 1a). In contrast, M1 (CD11b^+^/CD206^−^) macrophages decreased (from 14.17 to 7.71%), and the M1/M2 ratio in E-MNCs markedly decreased compared with that in PBMNCs (M1/M2; from 13.14 to 1.55) (Fig. 2c). Meanwhile, many cells in E-MNCs were also characterized by an increased fraction of CD3-positive T lymphocytes (Fig. 2b), and CXCR4^+^/CXCR6^−^ cells (as Th2 cells) proliferated from approximately 0.1 to 20% in CD3^+^/CD4^+^ T lymphocytes (Additional file 1b). However, there was no change in the cell number of Th1 and Th17 cells during 5G-culture (CXCR3^+^/CD3^+^/CD4^+^ cells and CXCR4^+^/CXCR6^+^/CD3^+^/CD4^+^ cells, respectively) (Additional file 1b). Consistent with this phenomenon, the inhibited mRNA expression of inflammatory genes (IL-1β and IFN-γ) and the upregulated expression of anti-inflammatory and vasculogenic genes (IL-10 and VEGF-A) were clearly seen (Fig. 2d). Additionally, when we evaluated the existence of a functional EPC fraction in E-MNCs, the number of EPC colonies derived from E-MNCs remarkably increased at day 7 after the plating of MNCs for EPC-CFA (Fig. 2e). These colonies were positive for ILB4-FITC (ILB4; binds to endothelial cells) and AcLDL-DiI (AcLDL; labels endothelial cells) (Additional file 1c), and endothelial stem/progenitor cells (c-Kit^+^/Sca-1^+^/lineage^−^) certainly increased by 5G-culture, though they were fairly few in number (approximately 2.5%) compared to other fractions such as macrophages or T lymphocytes (Additional file 1d). These phenomena strongly suggested that E-MNCs had acquired anti-inflammatory and vasculogenic characteristics during 5G-culture.

### Macroscopic observations after transplantation

**Fig. 3:**
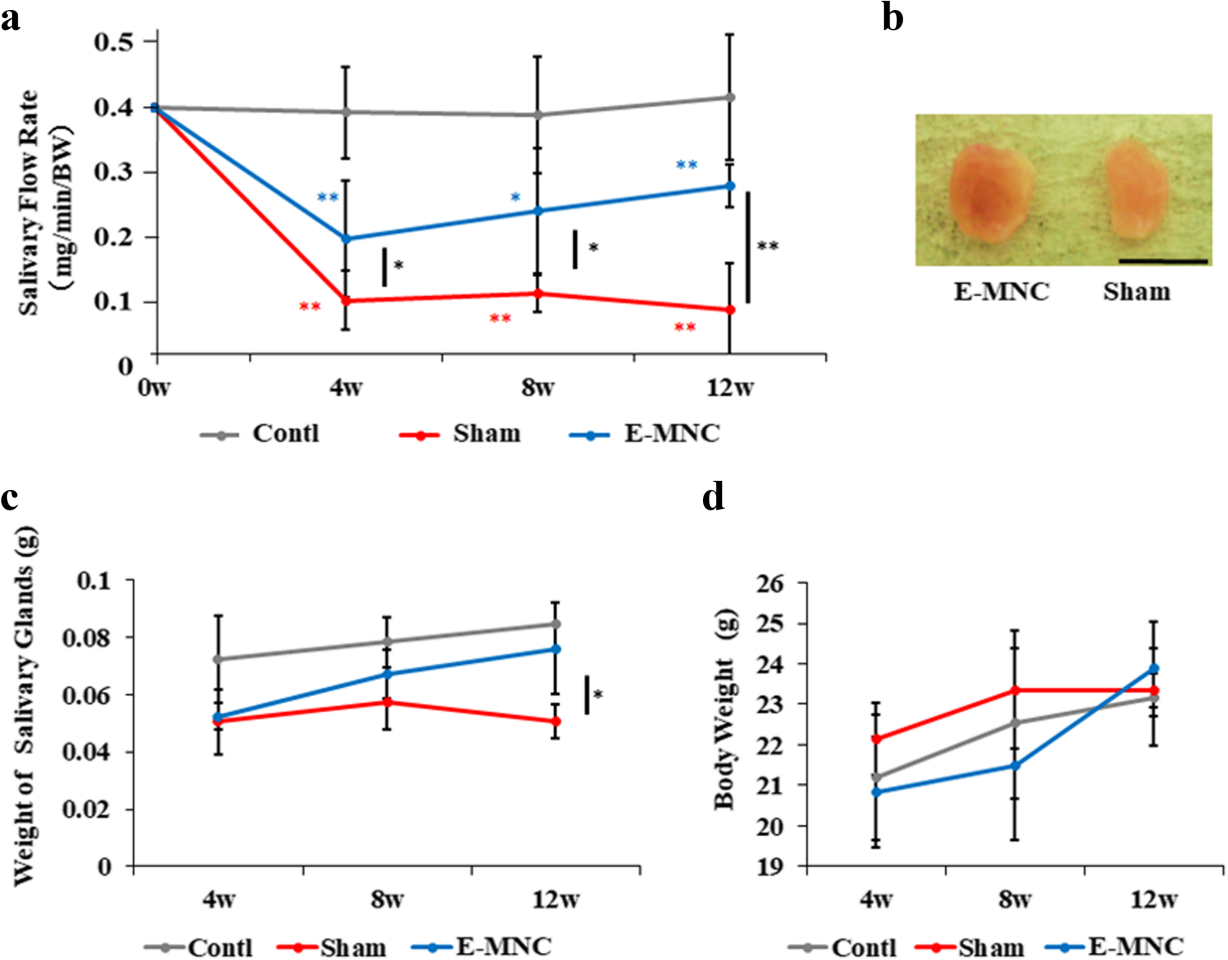
Macroscopic findings after IR and cell transplantation. **a** Changes of salivary flow rate (SFR) at 0, 4, 8, and 12 weeks after IR. **b** Gross appearance of harvested submandibular glands of the E-MNC group (EMNC-treated mice) and sham group (non-treated mice) at 12 weeks after IR (scale bar, 5 mm). **c** Weight changes of both submandibular glands at 4, 8, and 12 weeks after IR. **d** Changes of body weight at 4, 8, and 12 weeks after IR. The blue asterisk represents statistical significance between the control and E-MNC groups (**p < 0.01, *p < 0.05). The red asterisk represents statistical significance between the control and sham groups (**p < 0.01, *p < 0.05). The black asterisk represents statistical significance between the sham and E-MNC groups (**p < 0.01, *p < 0.05)

Overall, saliva secretions were increased in E-MNC-transplanted mice at weeks 4, 8 (*p < 0.05), and 12 (**p < 0.01) post-IR when compared to non-transplanted mice, and they gradually recovered after 4 weeks post-IR (Fig. 3a). Meanwhile, SFR in non- or PBMNC-transplanted mice was reduced severely after 4 weeks (Fig. 3a, Additional file 2a). In gross appearance, the size of submandibular glands harvested from E-MNC-transplanted mice was larger, and their appearance seemed to be usual when compared with those from non-treated mice at 12 weeks after IR (Fig. 3b). At 12 weeks after IR, the weights of the submandibular glands in E-MNC-treated mice had recovered when compared with those in non-treated mice (Fig. 3c; *p < 0.05). Although the body weights of mice increased gradually, here was no statistically significant difference between the groups (Fig. 3d).

### Detection of donor cells in the submandibular glands

**Fig. 4:**
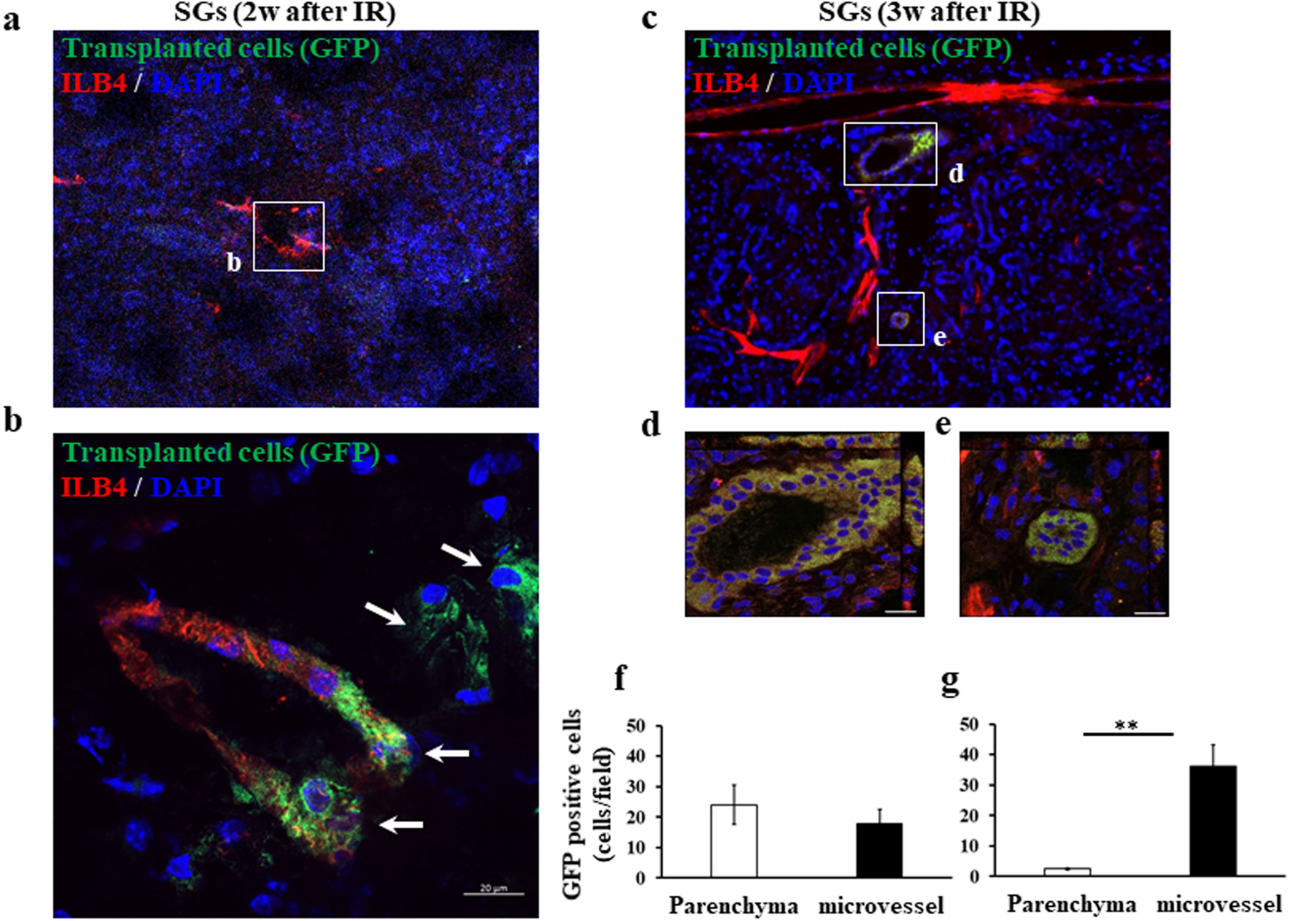
Detection of transplanted E-MNCs. **a** At 2 weeks after IR, GFP-expressing cells were detected in the microvessels and parenchyma (× 200) in the submandibular glands. The blood vessels were stained by ILB4. **b** The white box area in **a** was magnified (scale bar, 20 μm) (× 400). At 2 weeks, GFP-expressing cells (white arrow) were recognized clearly in vascular epithelia and parenchyma at the periphery of blood cells in the submandibular glands. Green, transplanted GFP-positive E-MNCs; red, blood vessels stained by ILB4; blue, DAPI. **c** At 3 weeks, they were detected only in the microvessels (scale bar, 200 μm) (× 200). **d**, **e** The white box areas in **c** were magnified (scale bar, 20 μm) (× 400). Green, transplanted GFP-positive E-MNCs; red, blood vessels stained by ILB4; blue, DAPI. **f**, **g** The graphs show the number of observed EGFP-expressing cells (× 200) in microvascular endothelium and perivascular parenchyma of gland tissues in E-MNC-transplanted mice at 2 weeks post-IR (**f**) and at 3 weeks post-IR (**g**)

At 2 weeks post-IR, some microvascular endothelial cells stained by ILB4-DyLight 594 (red) expressed EGFP (E-MNCs; green), and scattered EGFP-expressing cells were also seen at the perivascular parenchyma of gland tissues in E-MNC-transplanted mice (Fig. 4a, b). However, EGFP-expressing cells could be detected only in some microvessels and were absent in the parenchyma of E-MNC-transplanted submandibular glands at 3 weeks post-IR (Fig. 4c–e). Quantitative analysis revealed that some EGFP-expressing cells were observed among microvascular endothelial cells (about 18 cells/field) and in the perivascular parenchyma (about 24 cells/field) of gland tissues in E-MNC-transplanted mice at 2 weeks after IR (Fig. 4f). However, EGFP-expressing cells could be detected only in some microvessels (about 36 cells/field) and scarcely in the parenchyma (about 3 cells/field) at 3 weeks (Fig. 4g).

### Gene expressions and histological observations related to tissue regenerative activity from 4 to 12 weeks post-IR

**Fig. 5:**
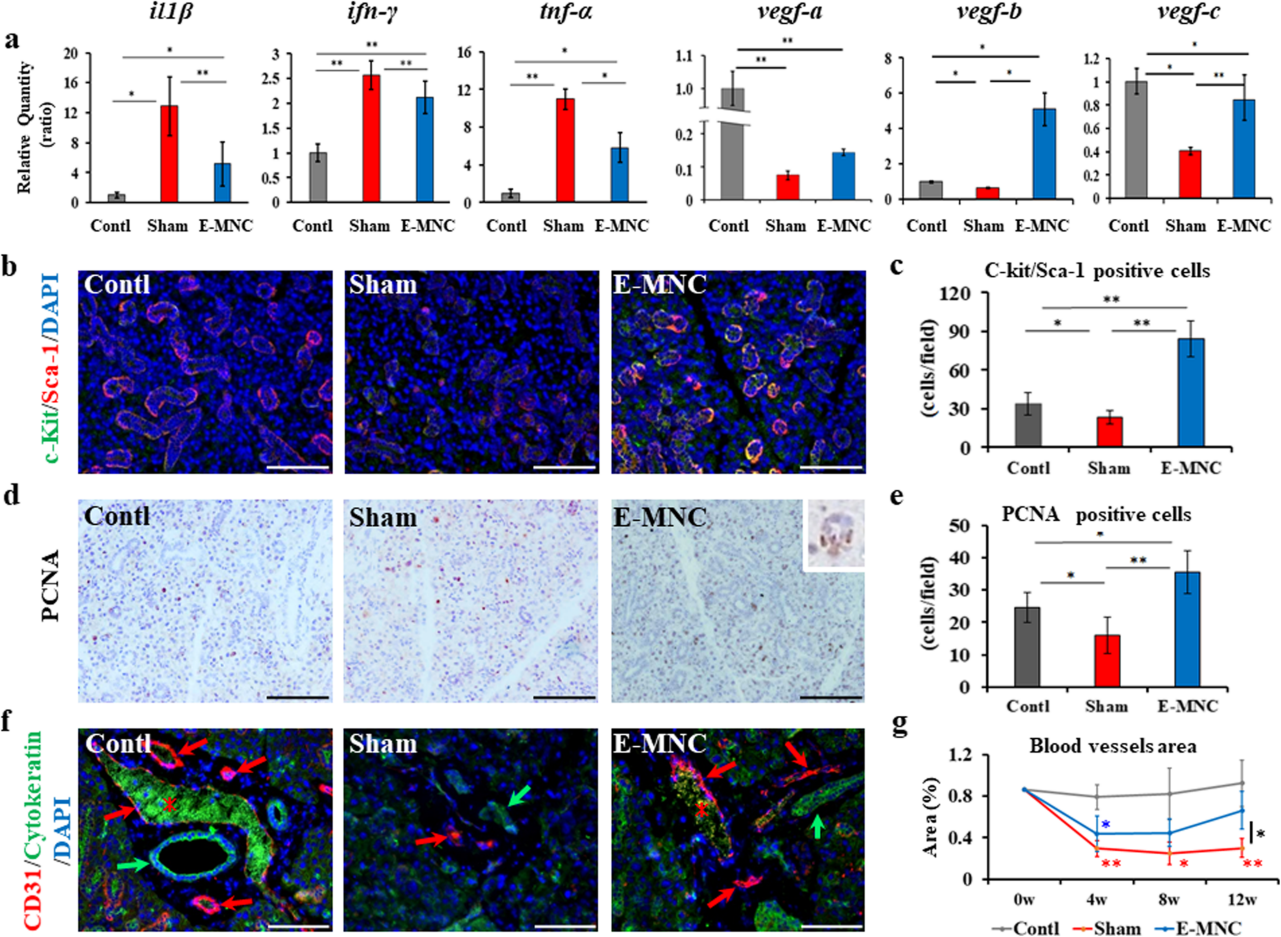
Gene expressions and histological findings at 4 to 12 weeks after IR. **a** mRNA expressions of inflammatory (IL-1β, IFN-γ, and TNF-α) and VEGF (A, B, and C) genes in the submandibular glands at 4 weeks post-IR (**p < 0.01, *p < 0.05). **b** Sca-1 and c-Kit expressions (red, Sca-1; green, c-Kit; scale bar, 50 μm) (× 200) at 4 weeks post-IR. **c** The graph shows the number of Sca-1/c-Kit double-positive cells in the submandibular glands (**p < 0.01, *p < 0.05). **d** Detection of PCNA (stained in brown; scale bar, 50 μm) (× 200) at 4 weeks post-IR. The graph shows the number of PCNA-positive cells in the submandibular glands (**p < 0.01, *p < 0.05). **e** The graph shows the number of PCNA-positive cells in the submandibular glands (**p < 0.01, *p < 0.05). **f** CD31 (red) and cytokeratin (green) immunostaining at 12 weeks after IR (scale bar, 50 μm) (× 200). Blue, DAPI; red arrow, blood vessels; green arrow, ducts; red asterisk, non-specific expression of blood cells (green expression in blood vessels). **g** Changes of the blood vessel area (%) of the salivary glands after 4, 8, and 12 weeks after IR. Blue asterisk represents statistical significance between the control and E-MNC groups (*p < 0.05). Red asterisk represents statistical significance between the control and sham groups (**p < 0.01, *p < 0.05). Black asterisk represents statistical significance between the sham and E-MNC groups (*p < 0.05)

At 4 weeks after IR, due to irradiation, mRNA expressions of inflammatory (IL-1β, IFN-γ, and TNF-α) genes in the submandibular glands of non- and E-MNC-transplanted mice were significantly upregulated when compared with those in the control group (Fig. 5a). However, the mRNA expressions in E-MNC-transplanted mice were significantly downregulated when compared with non-transplanted mice (IL-1β and INF-γ, **p < 0.01; TNF-α, *p < 0.05). The upregulation of vasculogenic (VEGF-A, VEGF-B, and VEGF-C) gene expressions was seen in the submandibular glands of E-MNC-transplanted mice when compared with non-transplanted mice (VEGF-B, *p < 0.05; VEGF-C, **p < 0.01) (Fig. 5a). The expressions of VEGF-A, VEGF-B, and VEGF-C in E-MNC-treated mice were upregulated by 1.9-, 7.8-, and 2.1-fold, respectively. Consistent with these results, the expression levels of Flt-1 (VEGFR-1) and Flt-4 (VEGFR-3) genes were significantly higher (Flt-1, 2.3-fold; Flt-4, 2.5-fold) in treated mice when compared with non-treated mice (Flt-1, *p < 0.05; Flt-4, *p < 0.05) (Additional file 2b). Meanwhile, there was no significant difference among the groups with regard to flk-1 (VEGFR-2) mRNA expression (Additional file 2b). As for histological observations, c-Kit and Sca-1 (markers for salivary progenitor cells) were prominently expressed in ductal cells of E-MNC-treated mice (Fig. 5b). The number of double-positive cells in E-MNC-transplanted mice was significantly larger than those of the control (**p < 0.01) and non-transplanted mice (**p < 0.01) (Fig. 5c). Brown-stained PCNA-positive cells were identified in acinar cells (box area in E-MNC specimen) of the submandibular glands in all groups (Fig. 5d), but the number of positive cells for PCNA was larger in E-MNC-treated mice than in control (*p < 0.05) and non-transplanted mice (**p < 0.01) (Fig. 5e). CD31 staining showed that irradiation remarkably decreased the blood vessels in the submandibular glands of non-treated mice until 12 weeks (Fig. 5f). Only the narrow and feeble blood vessels were detected in non-treated mice. In contrast, the areas of the blood vessels in E-MNC-treated mice were initially reduced by 4 weeks post-IR, but those areas gradually recovered by 12 weeks (Fig. 5g). At 12 weeks post-IR, the blood vessels in E-MNC-transplanted mice seemed to have been reconstructed markedly (Fig. 5f, g). Furthermore, regarding the saliva composition, the concentration of EGF, which plays an important physiological role in the maintenance of SG and oral mucosal tissues, in the saliva secreted from E-MNC-treated mice was markedly elevated when compared to that of non-transplanted mice (*p < 0.05) (Additional file 3a). This finding suggests the tissue regenerative activity was surely accelerated in E-MNC-treated SGs.

### Histological observations at 12 weeks post-IR

**Fig. 6:**
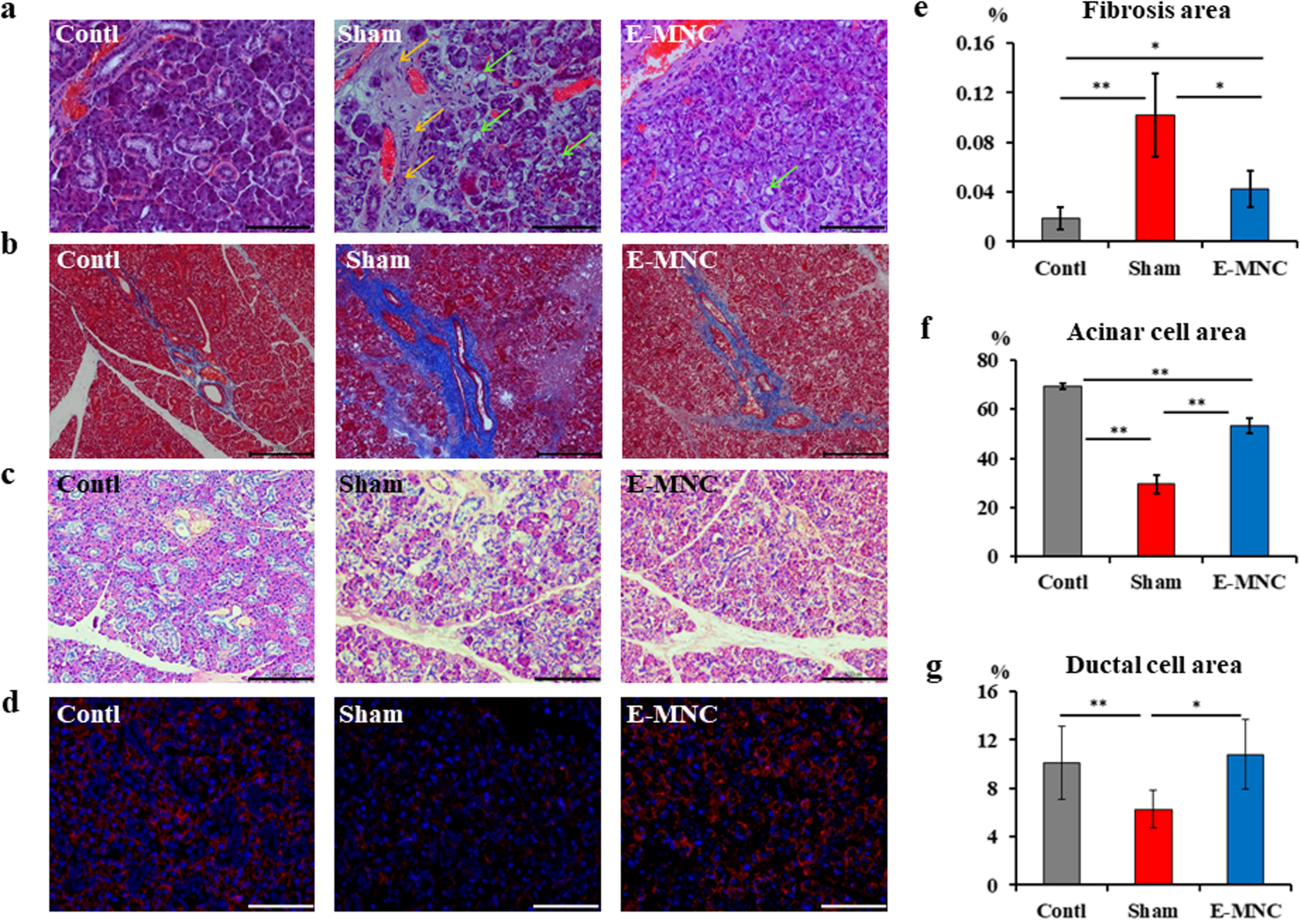
Histologic analysis of the submandibular glands at 12 weeks after IR. **a** Hematoxylin and eosin staining (scale bar, 50 μm) (× 200). Yellow arrow, fibrosis area; green arrow, vacuolar degeneration. **b** Masson’s trichrome staining (scale bar, 200 μm) (× 40). Fibrosis areas are stained blue. **c** PAS staining (scale bar, 100 μm) (× 100). Acinar cells were stained red. **d** Detection of the co-transporter for NKCC1 (a marker of acinar cells). Red, NKCC1; blue, DAPI (scale bar, 50 μm) (× 200). **e** The percentages of fibrosis area per field (%) (**p < 0.01, *p < 0.05). **f** The percentage area of acinar cells per field (%) (**p < 0.01). **g** The percentage area of ductal portions per field (%) (**p < 0.01, *p < 0.05)

At 12 weeks after IR, vanishing and vacuolar degeneration in the acinar cell area was seen in irradiated submandibular glands, particularly in non-transplanted mice (Fig. 6a). Furthermore, a remarkable fibrosis area was found in non-treated mice (Fig. 6a, b). However, the fibrosis in E-MNC-transplanted mice was visibly suppressed when compared to non-treated mice (Fig. 6b, e; *p < 0.05). Meanwhile, acinar cells decreased by irradiation in non-transplanted mice and E-MNC-treated mice, but PAS staining revealed that an approximately 1.8-fold larger area of acinar cells was preserved in E-MNC-treated mice compared to non-treated mice (Fig. 6c, f; **p < 0.01). Furthermore, the expression of NKCC1 (a marker of salivary acinar cells) in treated mice seemed to be the same level as in the control, but the level in non-treated mice was extremely low (Fig. 6d). This result was consistent with that of the PAS staining. Finally, the areas of ductal cells (stained by cytokeratin as shown in Fig. 5f) in the submandibular glands of non-treated mice were narrow (Fig. 6g). However, the percentage area in E-MNC-treated mice recovered to the same level as in control mice (Fig. 6g). Consistent with these observations, the expression level of AQP5 gene, which plays a role in the generation of saliva in SGs, was recovered in E-MNC-treated mice (**p < 0.01, *p < 0.05) (Additional file 3b). This result suggests E-MNC injection ameliorates not only tissue reconstruction but also the saliva secretory function at this stage.

## Discussion

In this study, we demonstrated that a cell therapy approach based on E-MNCs has therapeutic effects on radiogenic salivary hypofunction. The positive study outcomes were as follows: (1) treatment by E-MNCs, obtained from the peripheral blood, reliably recovered saliva secretion; (2) E-MNCs clearly promoted tissue regenerative activities in the atrophic glands via their anti-inflammation and vascularization actions as explants; and (3) E-MNCs might have affected these phenomena in a paracrine manner and/or by their vascular differentiation. These outcomes indicated that this strategy could be a promising option for developing future treatments.

As for the recovery of dysfunction, saliva secretion in the treated mice (E-MNC group) was reduced up to 4 weeks as with non-treated mice (IR group) (a 50% reduction in normal control mice), but less reduction was shown in treated mice when compared with non-treated mice (a 77% reduction). Then, saliva production gradually recovered in treated mice to a level of 35% less than that of normal mice at 12 weeks. In contrast, the reduction rate in non-treated mice (77–82% reduction) did not change during the evaluation period of this study. Consistent with this result, the submandibular glands in treated mice regained their weight to the level of those in normal mice at 12 weeks, but the weights were significantly less in non-treated mice. In addition, EGF concentration in the saliva secreted from the treated mice was elevated 3.2-fold higher than that from non-treated mice at 8 weeks. These phenomena definitely indicated that E-MNCs had a certain therapeutic effect in reducing radiogenic damage and/or restoring the secretory function of atrophic glands. Recently, proposed experimental approaches using SG stem cells, BMDCs, and MSCs have shown their ability to functionally restore SGs injured by irradiation [6, 9, 11]. However, as mentioned above, their actual availability is thought to be limited, because these autologous cells can only be harvested in a limited number from donor tissues. Furthermore, to obtain a sufficient number of capable stem cells, an expansion process is required in vitro, but the reality is that somatic stem cells such as MSCs lose their plasticity significantly during cell expansion and passaging, in particular depending on the patient’s age and morbidity [29–31]. The present study revealed that E-MNCs can rescue the dysfunction with high efficacy with a smaller number of transplanted cells (5 × 10^4^/gland). This outcome led us to believe that cell therapies using E-MNCs will be promising for future clinical applications because the peripheral blood is a readily accessible cell source that can be harvested with minimal invasiveness in elderly patients. Moreover, E-MNCs may contribute the functional restoration of atrophic glands more directly than BMDCs or MSCs, because exogenous MSCs can affect tissue regeneration by eliciting the polarization of macrophages toward an anti-inflammatory M2 phenotype, which produce large amounts of IL-10 [32, 33]. Indeed, E-MNCs can be expanded in primary culture, and the most significant advantage is that this functional primary culture system enhances the EPC fraction and elicits the regenerative phenotype of macrophages and T lymphocytes from PBMNCs. In this study, mouse PBMNCs were composed of approximately 46% lymphocytes (24% CD3^+^ T lymphocytes and 22% B220^+^ B lymphocytes), approximately 15% monocytes/macrophages (14% CD11b^+^/CD206^−^ M1 macrophages and 1% CD11b^+^/CD206^+^ M2 macrophages), and approximately 0.02% endothelial stem/progenitor cells (c-Kit^+^/Sca-1^+^/lineage^−^). However, after 5G-culture, E-MNCs were comprised of approximately 66% lymphocytes (47% CD3^+^ T lymphocytes and 19% B220^+^ B lymphocytes), approximately 13% monocytes/macrophages (7% CD11b^+^/CD206^−^ M1 macrophages and 6% CD11b^+^/CD206^+^ M2 macrophages), and approximately 2.5% endothelial stem/progenitor cells (c-Kit^+^/Sca-1^+^/lineage^−^). Hence, while colony-forming EPCs were significantly increased, definitive M2 macrophages (an increase of approximately fivefold) appeared after 5 days of this culture, and the ratio of T lymphocytes also increased to approximately twofold. These T lymphocytes were considered to support the anti-inflammatory phenotypes of cells, because M2 macrophages were induced by the co-existence of these T lymphocytes, and IL-10 mRNA expression was significantly upregulated in E-MNCs. In fact, only the CXCR4^+^/CXCR6^−^ Th2 cell fraction increased in CD3^+^/CD4^+^ T helper cells after 5G-culture. In contrast, the expressions of proinflammatory genes such as IL-1β and IFN-γ were largely downregulated. It has been reported that the M2 type of macrophages contributes to the inactivation of inflammation and fibrosis regression when an injury stabilizes [34, 35]. Therefore, even with a cell number of approximately 1% of BMDC transplantation [6], those cell populations in E-MNCs may have been able to restore the atrophic glands. Moreover, several studies have shown the effectiveness of BMDC or cultured MSC therapies via local and systemic routes on the atrophic disease of the salivary glands [6, 8, 9. 12, 14, 15]. Among these studies, Xu et al. have demonstrated the immunologic regulatory functions of umbilical cord MSCs play an important role in the amelioration of Sjögren’s syndrome on both of basic studies and phase 1/2 clinical trials [36]. Considering such functional mechanisms of MSCs, effective cell populations in E-MNC may display the therapeutic functions through both of local and systemic injections for atrophic glandular diseases due to not only the radiation therapy but also Sjögren’s syndrome.

With regard to the promotion of regenerative activities in the atrophic glands, transplanted E-MNCs must have functioned mostly in revascularization. Although the exact mechanisms by which they enhanced regeneration were not well elucidated in this study, the fact that microvascular endothelial cells in SGs are targets for radiation damage led us to this assumption [6, 37]. 5G-culture of PBMNCs increased the number of colony-forming EPCs and M2 macrophages, which are known to play particularly important roles in vascularization by localizing themselves nearby newly forming blood vessels and aiding in their stabilization and fusion [24–26]. As noted in these prior works, EGFP-labeled donor E-MNCs are detectable in portions of both the vascular endothelium and perivascular gland tissues at 2 weeks post-IR. In addition, CD3^+^ T lymphocytes, which are concentrated in E-MNCs with the anti-inflammatory phenotype, must be able to support the vasculogenic functions of EPCs and M2 macrophages because IL-10 and VEGF are produced by their interaction. Our in vivo data revealed elevated gene expression of VEGFs and their receptors accompanied by the downregulation of anti-inflammatory gene expressions in E-MNC-treated SGs at 4 weeks post-IR. In particular, VEGFR-3 (also known as Flt-4), which contributes to angiogenic sprouts [38], was significantly upregulated in treated SGs. VEGFR-3 is known to augment VEGF-induced angiogenesis and sustained angiogenesis even in the presence of VEGFR-2 (also known as Flk-1) inhibitors [39]. Moreover, Tammela et al. demonstrated that VEGFR-3 is strongly expressed in the angiogenic vessel front during the early postnatal period, but not in mature vessels [39]. Taking these data together, transplanted E-MNCs should have ameliorated chronic inflammation and subsequently at least induced revascularization. This led us to hypothesize that vasculogenic paracrine effects were accelerated forcefully by anti-inflammatory phenotypes of macrophages and T lymphocytes in E-MNCs. Indeed, we found that acinar and ductal cells clearly proliferated with less fibrosis in treated mice at 12 weeks. Then, the upregulation of AQP5 gene (a functional marker of SGs) in such specimens was also seen. Previous studies using modified or depleted macrophages have pointed to a role for them in inhibiting the fibrotic response [34, 40, 41]. On the other hand, it is known that the macrophage phenotype readily changes based on the spatiotemporal conditions at injured sites [26]. Therefore, transplantation of heterogenous cell fractions in E-MNCs, which are composed of M1 and M2 macrophages, lymphocytes in the anti-inflammatory phase, and endothelial stem/progenitor cells, might have the advantage of being largely unaffected by undesirable host circumstances. At any rate, these vasculogenic and anti-inflammatory effects by E-MNCs may protect against fibrosis and then contribute to SG cell proliferation, even in severe radiogenic atrophic glands.

## Conclusions

In conclusion, our results demonstrated that cell-based therapy using 5G-cultured PBMNCs can be effectively employed for radiation-induced salivary gland dysfunction without the need for complicated procedures [39]. This strategy can be easily applied in clinical settings, has low invasiveness, and uses a readily available source of cells. However, due to their vasculogenic properties, the transplantation of these cells may activate tumor proliferation, angiogenesis, or metastasis [42]. Therefore, the application of our current strategy should be restricted to patients with complete remission following radiation therapy for head and neck cancer. Further investigations are needed to understand the exact mechanisms of atrophic tissue restoration by transplanted E-MNCs and to determine the practical usefulness of this strategy for future clinical applications. Another limitation is that this study used a single-dose irradiation and may not correlate with current therapeutic regimens. Therefore, future investigation should be carried out by using additional clinical models such as fractionated-dose irradiation with chemotherapy. This study proposes that the serum-free 5G-culture system can make the peripheral blood a highly useful cell source for atrophic salivary gland regeneration.

## Supplementary information

**Additional file 1. **a Flow cytometric analysis of M2 macrophages (CCR2^-^/Galectin3^+^) in the PI^-^/CD11b^+^ cell fraction of PBMNCs (Day 0) and E-MNCs (Day 5). CCR2^-^/Galectin3^+^ cells occupied approximately 50% of the CD11b positive cell fraction in E-MNCs. High levels of intracellular galectin 3 expression are considered essential for transcriptional activation towards M2 macrophages after M1. b Flow cytometric analysis of T helper cells (CD3^+^/CD4^+^) in the PI fraction of PBMNCs (Day 0) and E-MNCs (Day 5). CXCR4^+^/CXCR6^−^ cells (Th2) were approximately 20% of the CD3- and CD4-positive T helper cell fraction of E-MNCs. Th1, CXCR3 positive cells in T helper cells; Th17, CXCR4 and CXCR6 positive cells in T helper cells. **c** Representative pictures of EPC-CFU (Scale bar: 100 μm) at 7 days of EPC-CFA (100×), and the right panel shows ILB4-conjugated FITC binding and AcLDL-DiI uptake of each EPC-CFU (Scale bar: 100 μm) (40×). d Percentage of endothelial stem/progenitor cell fraction (c-kit^+^/Sca-1^+^/lineage^−^) in PBMNCs and E-MNCs.

**Additional file 2. **a Changes of salivary flow rate (SFR) in sham and PBMNCs groups at 0, 4, 8, and 12 weeks after IR. b mRNA expressions of VEGFRs (flk1, flt-1, and flt4) at 4 weeks post-IR (**p < 0.01, *p < 0.05).

**Additional file 3.** a Concentration of EGF in saliva at 8 weeks after IR. The saliva secreted from E-MNC-treated mice was increased in EGF when compared to non-transplanted mice (*p < 0.05). b mRNA expressions of AQP5 at 12 weeks post-IR (**p < 0.01, *p < 0.05).

## Data Availability

All data generated or analyzed during the current study are included in this published article.

## Abbreviations

AcLDL: Acetylated low-density lipoprotein
AQP5: Aquaporin 5 which is a water channel protein
BM: Bone marrow
BMDCs: Bone marrow-derived cells
BSA: Bovine serum albumin
CFA: Colony-forming assay
CFU: Colony-forming units
DAPI: 4′,6-Diamidino-2′-phenylinodole
EDTA: Ethylenediaminetetraacetic acid
EGF: Epidermal growth factor
EGFP: Enhanced green fluorescent protein
E-MNCs: Effectively conditioned PBMNCs
EPCs: Endothelial progenitor cells
Flk: Fetal liver kinase
Flt: Fms-related tyrosine kinase
H&E: Hematoxylin and eosin
IFN-γ: Interferon-γ
IL-10: Interleukin-10
IL-1β: Interleukin-β
ILB4: Isolectin B4
IMDM: Iscove’s modified Dulbecco’s medium
IMRT: Intensity-modulated radiation therapy
IR: Irradiation
MSCs: Mesenchymal stem cells
PAS staining: Periodic acid-Schiff staining
PB: Peripheral blood
PBMNCs: Peripheral blood mononuclear cells
PBS: Phosphate-buffered saline
PCNA: Proliferating cell nuclear antigen
PFA: Paraformaldehyde
PI: Propidium iodide
qPCR: Quantitative real-time polymerase chain reaction
QQ-culture: Quality and quantity-controlled culture
SFR: Salivary flow rate
SG: Salivary gland
TNF-α: Tumor necrosis factor-α
VEGF: Vascular endothelial growth factor
VEGFR: Vascular endothelial growth factor receptor
