# Supplementary figures and images for: Anti-inflammatory and vasculogenic conditioning of peripheral blood mononuclear cells reinforces their therapeutic potential for radiation-injured salivary glands
Source: Stem Cell Res Ther. 2019 Oct 17;10:304. doi: 10.1186/s13287-019-1414-7 (PMC6798785; doi:10.1186/s13287-019-1414-7)

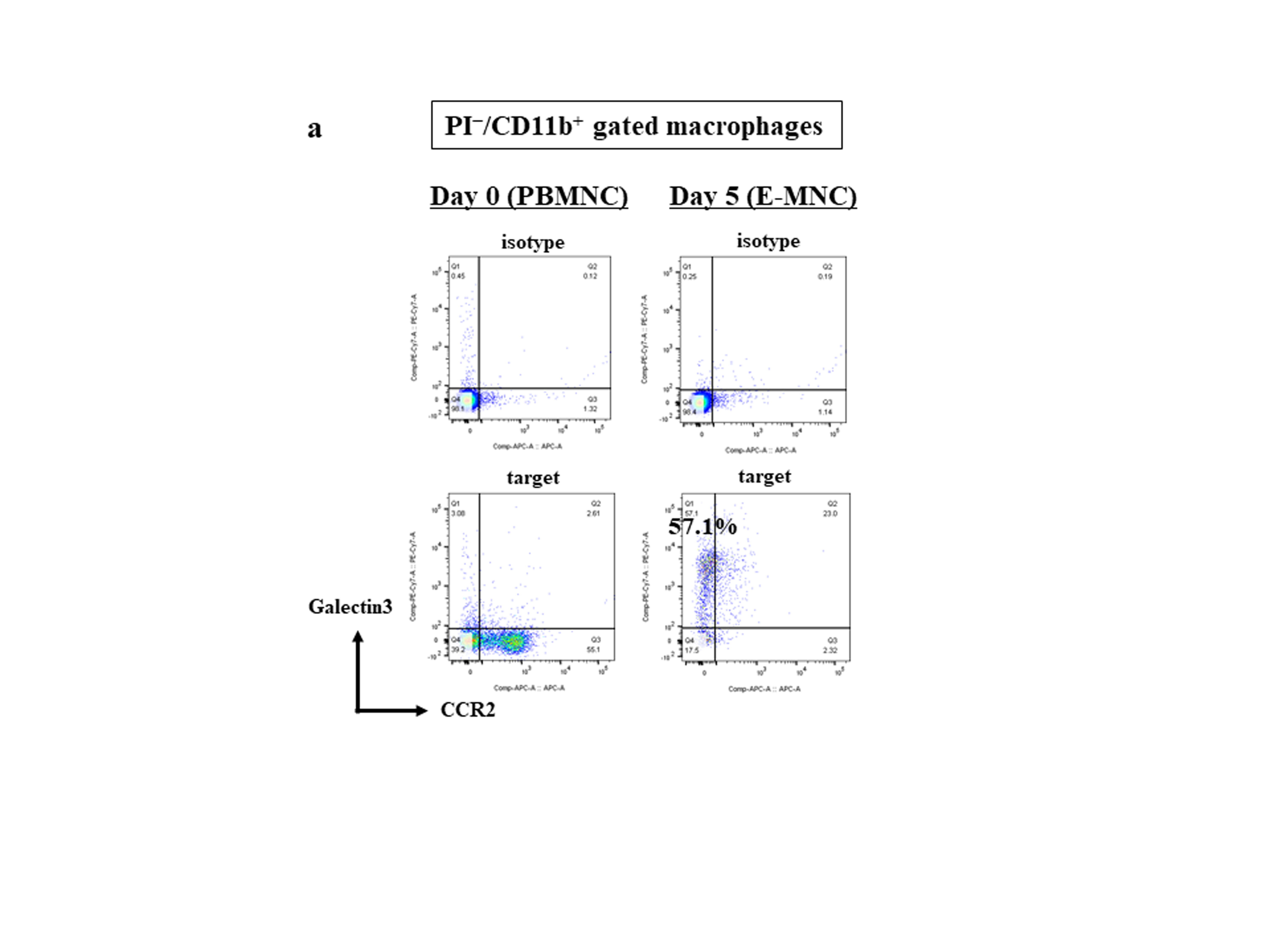

Supplement: Supplementary file 1 — Additional file 1. a Flow cytometric analysis of M2 macrophages (CCR2-/Galectin3+) in the PI-/CD11b+ cell fraction of PBMNCs (Day 0) and E-MNCs (Day 5). CCR2-/Galectin3+ cells occupied approximately 50% of the CD11b positive cell fraction in E-MNCs. High levels of intracellular galectin 3 expression are considered essential for transcriptional activation towards M2 macrophages after M1. b Flow cytometric analysis of T helper cells (CD3+/CD4+) in the PI fraction of PBMNCs (Day 0) and E-MNCs (Day 5). CXCR4+/CXCR6− cells (Th2) were approximately 20% of the CD3- and CD4-positive T helper cell fraction of E-MNCs. Th1, CXCR3 positive cells in T helper cells; Th17, CXCR4 and CXCR6 positive cells in T helper cells. c Representative pictures of EPC-CFU (Scale bar: 100 μm) at 7 days of EPC-CFA (100×), and the right panel shows ILB4-conjugated FITC binding and AcLDL-DiI uptake of each EPC-CFU (Scale bar: 100 μm) (40×). d Percentage of endothelial stem/progenitor cell fraction (c-kit+/Sca-1+/lineage−) in PBMNCs and E-MNCs. [file 13287_2019_1414_MOESM1_ESM.zip › Additional file 1-1.tif]

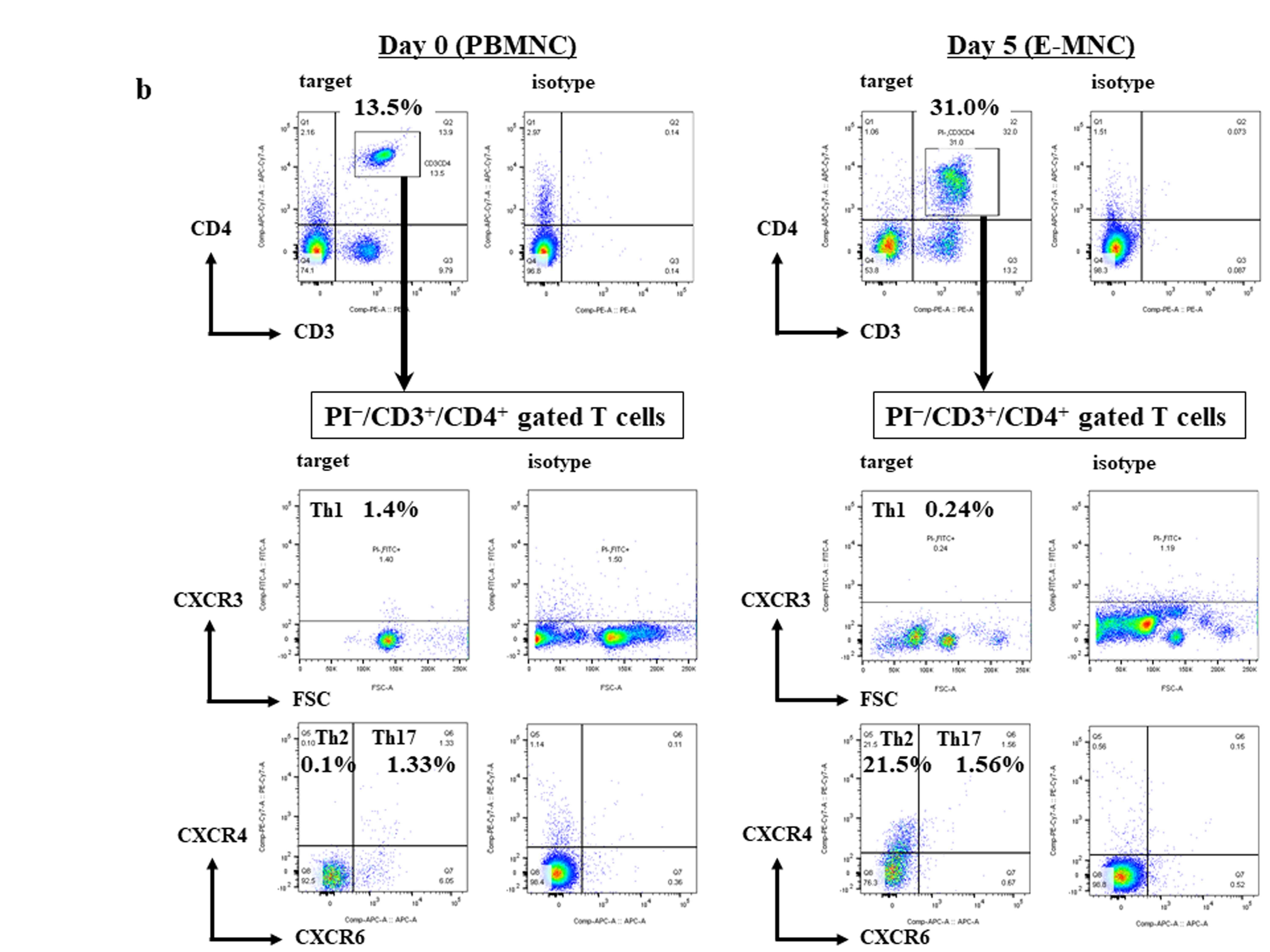

Supplement: Supplementary file 1 — Additional file 1. a Flow cytometric analysis of M2 macrophages (CCR2-/Galectin3+) in the PI-/CD11b+ cell fraction of PBMNCs (Day 0) and E-MNCs (Day 5). CCR2-/Galectin3+ cells occupied approximately 50% of the CD11b positive cell fraction in E-MNCs. High levels of intracellular galectin 3 expression are considered essential for transcriptional activation towards M2 macrophages after M1. b Flow cytometric analysis of T helper cells (CD3+/CD4+) in the PI fraction of PBMNCs (Day 0) and E-MNCs (Day 5). CXCR4+/CXCR6− cells (Th2) were approximately 20% of the CD3- and CD4-positive T helper cell fraction of E-MNCs. Th1, CXCR3 positive cells in T helper cells; Th17, CXCR4 and CXCR6 positive cells in T helper cells. c Representative pictures of EPC-CFU (Scale bar: 100 μm) at 7 days of EPC-CFA (100×), and the right panel shows ILB4-conjugated FITC binding and AcLDL-DiI uptake of each EPC-CFU (Scale bar: 100 μm) (40×). d Percentage of endothelial stem/progenitor cell fraction (c-kit+/Sca-1+/lineage−) in PBMNCs and E-MNCs. [file 13287_2019_1414_MOESM1_ESM.zip › Additional file 1-2.tif]

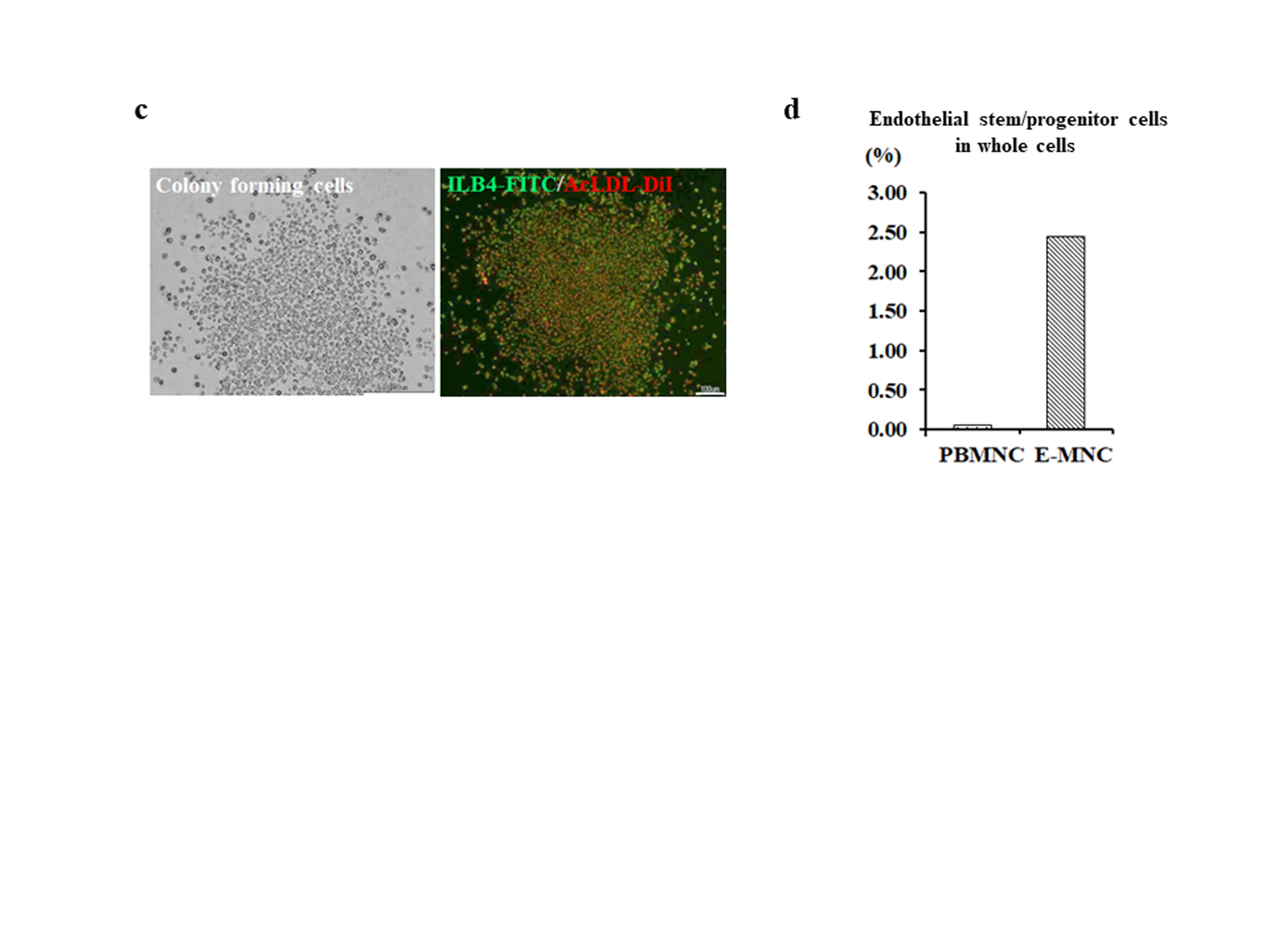

Supplement: Supplementary file 1 — Additional file 1. a Flow cytometric analysis of M2 macrophages (CCR2-/Galectin3+) in the PI-/CD11b+ cell fraction of PBMNCs (Day 0) and E-MNCs (Day 5). CCR2-/Galectin3+ cells occupied approximately 50% of the CD11b positive cell fraction in E-MNCs. High levels of intracellular galectin 3 expression are considered essential for transcriptional activation towards M2 macrophages after M1. b Flow cytometric analysis of T helper cells (CD3+/CD4+) in the PI fraction of PBMNCs (Day 0) and E-MNCs (Day 5). CXCR4+/CXCR6− cells (Th2) were approximately 20% of the CD3- and CD4-positive T helper cell fraction of E-MNCs. Th1, CXCR3 positive cells in T helper cells; Th17, CXCR4 and CXCR6 positive cells in T helper cells. c Representative pictures of EPC-CFU (Scale bar: 100 μm) at 7 days of EPC-CFA (100×), and the right panel shows ILB4-conjugated FITC binding and AcLDL-DiI uptake of each EPC-CFU (Scale bar: 100 μm) (40×). d Percentage of endothelial stem/progenitor cell fraction (c-kit+/Sca-1+/lineage−) in PBMNCs and E-MNCs. [file 13287_2019_1414_MOESM1_ESM.zip › Additional file 1-3.tif]

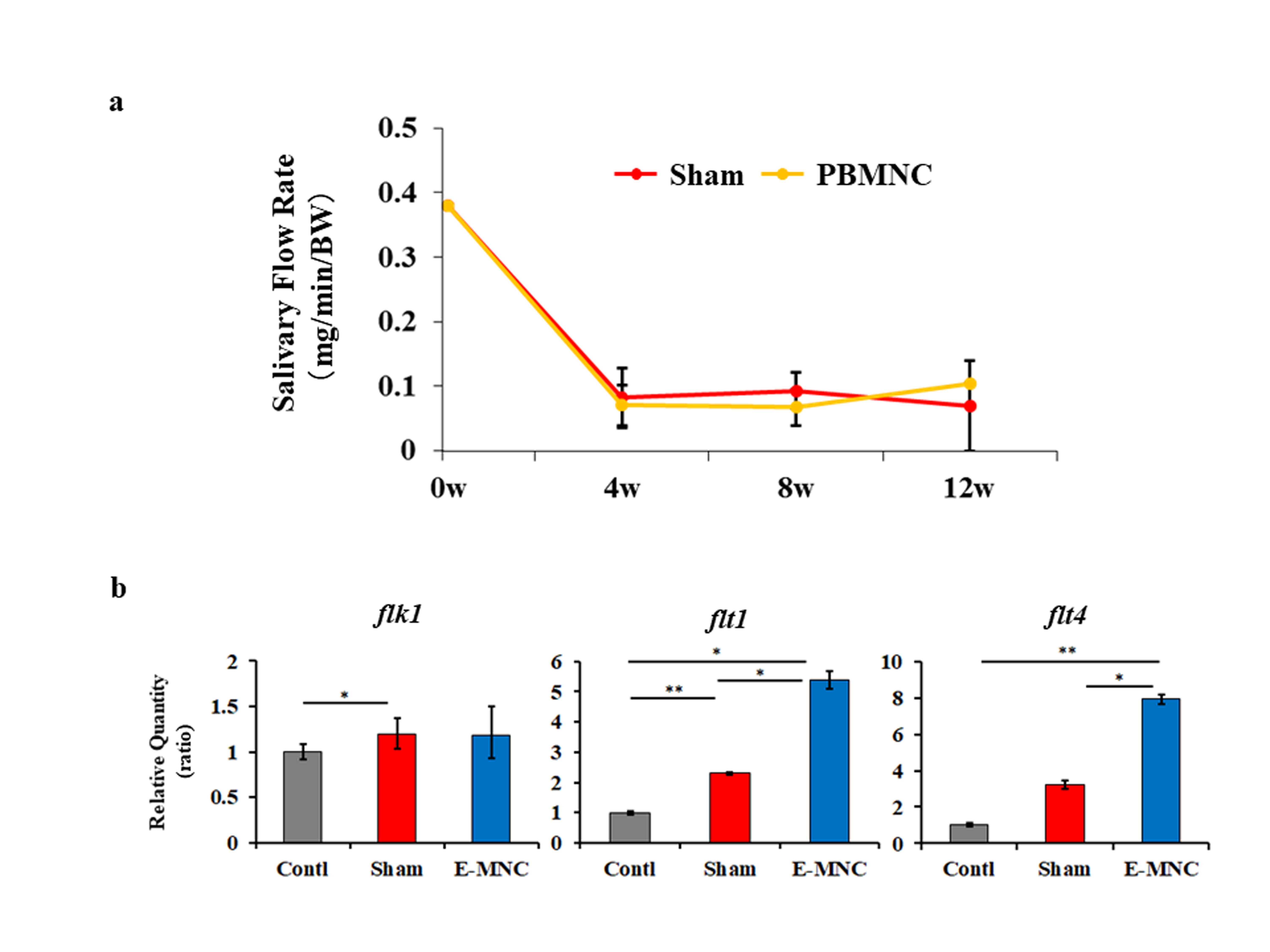

Supplement: Supplementary file 2 — Additional file 2. a Changes of salivary flow rate (SFR) in sham and PBMNCs groups at 0, 4, 8, and 12 weeks after IR. b mRNA expressions of VEGFRs (flk1, flt-1, and flt4) at 4 weeks post-IR (**p < 0.01, *p < 0.05). [file 13287_2019_1414_MOESM2_ESM.tif]

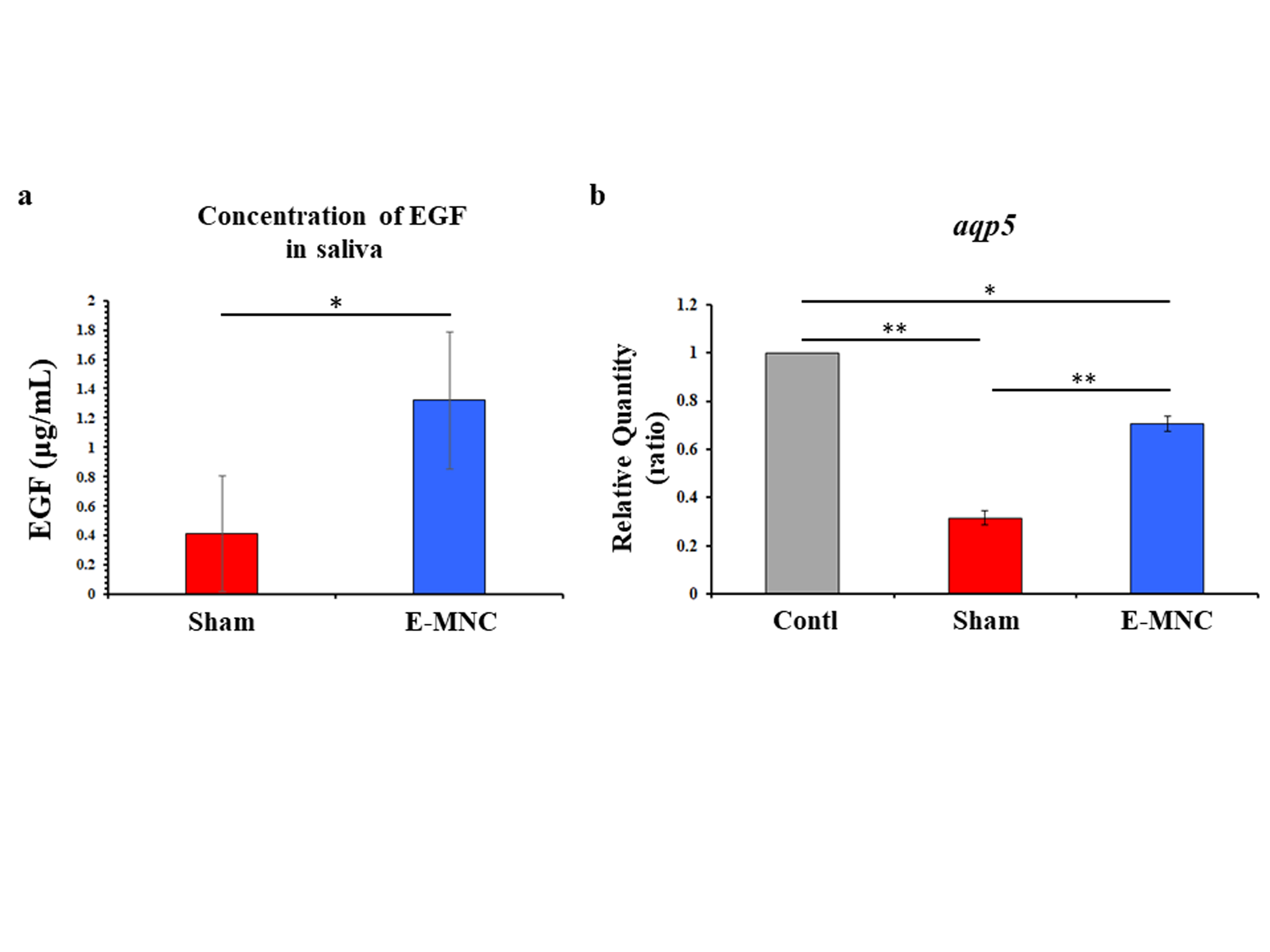

Supplement: Supplementary file 3 — Additional file 3. a Concentration of EGF in saliva at 8 weeks after IR. The saliva secreted from E-MNC-treated mice was increased in EGF when compared to non-transplanted mice (*p < 0.05). b mRNA expressions of AQP5 at 12 weeks post-IR (**p < 0.01, *p < 0.05). [file 13287_2019_1414_MOESM3_ESM.tif]
